# Barriers and enablers to sustaining self-management behaviours after completing a self-management support intervention for type 2 diabetes: a protocol for a systematic review and qualitative evidence synthesis

**DOI:** 10.12688/hrbopenres.13466.1

**Published:** 2021-12-14

**Authors:** Márcia Carvalho, Pauline Dunne, Dominika Kwasnicka, Molly Byrne, Jenny McSharry

**Affiliations:** 1 Health Behaviour Change Research Group, School of Psychology, National University of Ireland, Galway, Galway, Ireland; 2 School of Agriculture and Food Science, University College Dublin, Dublin, Ireland; 3 Faculty of Psychology, SWPS University of Social Sciences and Humanities, Aleksandra Ostrowskiego, Wrocław, Poland; 4 NHMRC CRE in Digital Technology to Transform Chronic Disease Outcomes, Melbourne School of Population and Global Health, University of Melbourne, Melbourne, Melbourne, Australia

**Keywords:** type 2 diabetes, self-management, self-management support interventions, behaviour change, behaviour maintenance, sustained behaviour change, systematic review, qualitative evidence synthesis

## Abstract

**Background: **Attendance at self-management support interventions is associated with improved outcomes for people with type 2 diabetes. However, initial improvements are often not sustained beyond one year, which may be a result of difficulties in sustaining positive changes made to self-management behaviours. The aim of this systematic review is to synthesise qualitative research on the barriers and enablers to sustaining self-management behaviours following completion of a self-management support intervention for type 2 diabetes.

Methods: The review will use the “best fit” framework synthesis method to develop a new conceptual model of sustained behaviour change in type 2 diabetes. MEDLINE (Ovid), EMBASE (Elsevier), CINAHL (EBSCO), PsycINFO (Ovid), SCOPUS, ProQuest Dissertations and Theses, WorldCat and Open Grey will be searched to identify primary qualitative studies. A parallel search will be conducted in Google Scholar to identify relevant theories for the development of an *a priori* framework to synthesise findings across studies. Methodological limitations of included studies will be assessed using an adapted version of the Critical Appraisal Skills Programme tool for Qualitative Studies. A sensitivity analysis will be conducted to examine the impact of studies with methodological limitations on synthesis findings. Confidence in the synthesis findings will be assessed using the GRADE-CERQual tool. Screening, data extraction, methodological limitation assessment, synthesis and GRADE-CERQual assessment will be conducted by one author with a second author independently verifying a randomly selected 20% sample.

**Discussion:** This review will develop a new model of sustained behaviour change in type 2 diabetes self-management. The findings can be used to inform the development of new interventions or revision of existing interventions to better support sustained engagement in type 2 diabetes self-management behaviours.

## Introduction

Type 2 diabetes is a progressive, chronic metabolic disease characterised by beta-cell dysfunction and insulin resistance ^1, 2^. The prevalence of type 2 diabetes and associated health and economic burden is rising worldwide ^1^. Approximately 462 million people (6.28% of the world’s population) live with type 2 diabetes worldwide ^3^, with this number expected to increase over the coming years ^1, 3^. Without adequate management, type 2 diabetes is associated with microvascular and macrovascular health complications, such as cardiovascular disease, blindness, neuropathy, kidney failure, and lower-limb amputation, and an increased risk of premature death and morbidity ^1, 2^. Achieving good glycaemic control through appropriate self-management is critical to prevent the progression of the disease and avoid health complications ^4, 5^.

Self-management is a broad concept encompassing all cognitive and emotional self-regulatory processes and behaviours an individual needs to perform to manage the physical and psychosocial consequences of living with type 2 diabetes ^6, 7^. Self-management of type 2 diabetes can be complex and demanding, as it can require significant lifestyle changes (i.e., diet and physical activity) and involves multiple self-management behaviours, such as medication taking and blood glucose monitoring, which individuals need to implement and sustain in their daily lives ^4, 8^. As a result, many people struggle to achieve and sustain optimal glycaemic management. Real-world evidence of patient profiles and diabetes care practices in developed countries demonstrate that less than 20% of people with type 2 diabetes achieve target blood glucose levels (<53 mmol/mol [<7%]) ^9, 10^.

Self-management support interventions aimed at assisting individuals in self-managing their condition are therefore a central component of type 2 diabetes care ^11, 12^. Attendance at self-management support interventions is recommended internationally for people with type 2 diabetes ^1, 11, 13^ and a wide range of self-management support interventions have been developed and implemented ^4, 8, 14, 15^. Although self-management support interventions vary in terms of mode of delivery, duration, intensity, type of provider, and content ^8, 15^, in general, interventions focus on one or any combination of the following components: education (providing information and developing self-management skills such as blood glucose monitoring), lifestyle (promoting and supporting changes in health behaviours relevant to type 2 diabetes, such as diet and physical activity), and psychosocial aspects (promoting and supporting the development of psychosocial skills to facilitate coping and management) ^8, 15^.

Several randomised controlled trials and systematic reviews indicate benefits from attendance at type 2 diabetes self-management interventions, such as improved biomedical (e.g., weight and glycaemic control), behavioural (e.g., dietary management, and physical activity), and psychosocial (e.g., diabetes knowledge, and quality of life) outcomes ^4, 14, 16, 17^. However, long-term follow-ups tend to show that while improvements in psychosocial outcomes are maintained, frequently people experience a decline in glycaemic management over time, particularly from six months to one-year post-intervention ^14, 17, 18^. Although the reasons for this decline in glycaemic management are poorly understood ^14, 18^, challenges in sustaining positive changes made to self-management behaviours are assumed to be an underlying cause ^14, 15^.

For the purpose of this review, in line with a published definition ^19^, the term sustained behaviour change is used to describe the continuous performance of self-management behaviours following an initial intentional change (during intervention) at a level that significantly differs from the baseline performance (pre-intervention) in the intended direction. Although there is lack of consensus on the timeframe used in defining sustained health behaviour change with definitions ranging from three-months to one-year post-intervention ^20^, the criterion of at least 3 months post-intervention will be used in this review.

To design interventions that effectively support sustained engagement in type 2 diabetes self-management behaviours, it is necessary to identify factors that influence the maintenance of self-management behaviours following completion of a self-management support intervention. A number of primary qualitative studies have been undertaken to explore the experiences of self-management of people with type 2 diabetes post-intervention ^21– 25^. However, to our best knowledge, available evidence has not yet been synthesised. Individual qualitative studies offer important insights into the individual’s experiences and perceptions and perceived barriers and enablers, but a synthesis of qualitative literature can facilitate the development of overarching insights that go beyond individual study findings ^26^.

Therefore, the main aim of this systematic review is to synthesise qualitative research on barriers and enablers to sustaining self-management behaviours following completion of a self-management support intervention for type 2 diabetes.

## Methods

This protocol has been prospectively registered on the International Prospective Register of Systematic Reviews (PROSPERO) to ensure the transparency of the research process (CRD42021281374). This systematic review and qualitative evidence synthesis protocol is reported in line with the Preferred Reporting Items for Systematic Reviews and Meta-Analyses Protocol (PRISMA-P) ^27^ (See *Extended data*: Supplementary File 1 ^28^).

The “best fit” framework synthesis method will be used ^29^. This is a flexible, transparent, and pragmatic method that builds on one or more existing theories to develop a new context-specific conceptual model to explain or describe a health behaviour ^29^. The choice of this analytical approach was informed by the RETREAT (Review question – Epistemology – Time/Timescale – Resources – Expertise – Audience and purpose – Type of Data) framework, which offers a criterion-based approach to guide the selection of most appropriate analytical approach for a review ^30^ (See *Extended data*: Supplementary File 2 ^28^).

**Figure 1.**
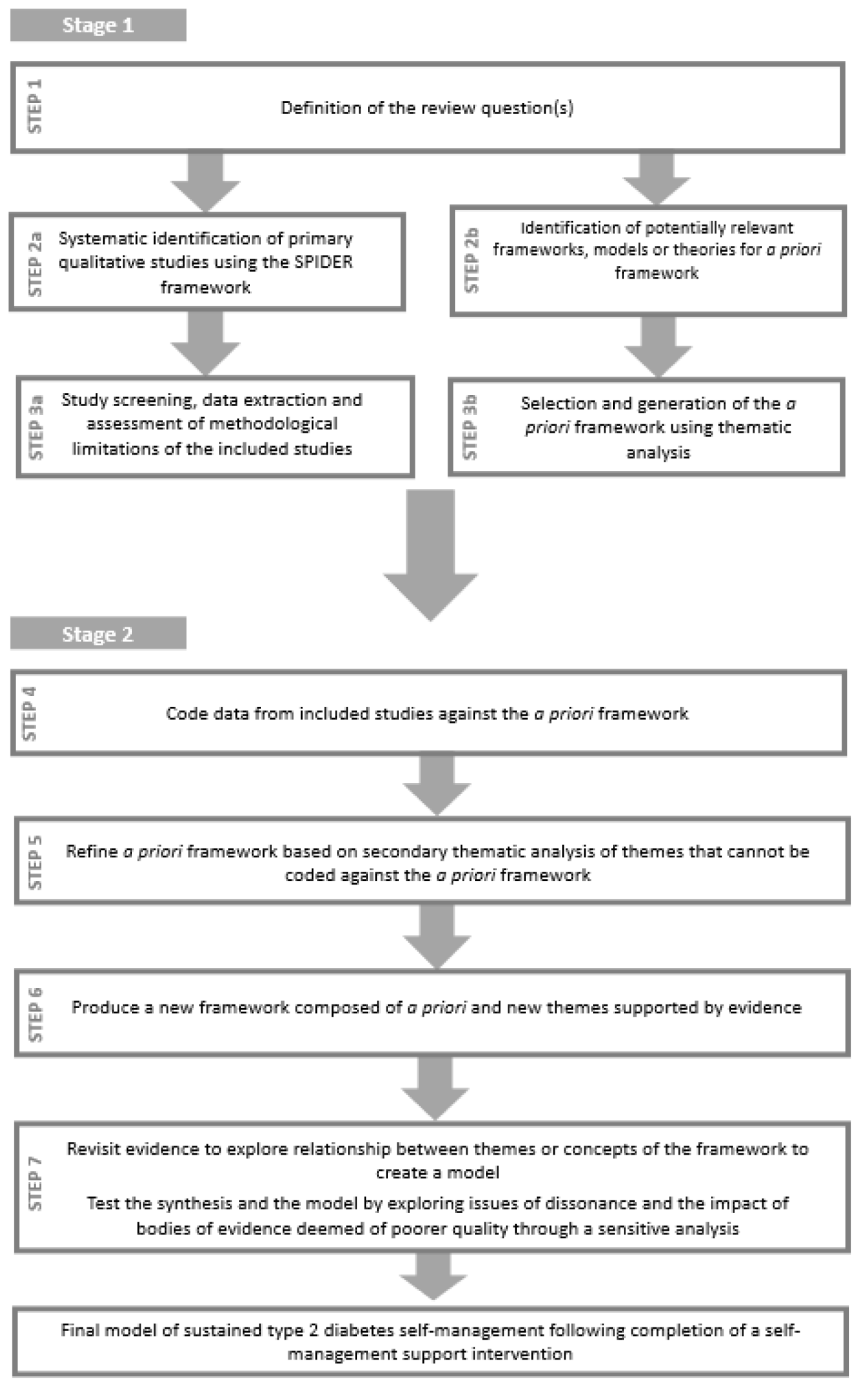
Two-stage review design (based on 29).

The “best fit” framework synthesis method involves the identification of a foundation theory or theories referred to as the *a priori* framework, the coding of the data from the primary studies included in the review against this *a priori* framework, and the secondary thematic analysis of the data that do not fit into the *a priori* framework ^29^. The process includes seven steps and can be conceptualised as divided into two stages (See Figure 1).

In the first stage, the review question(s) is determined and the primary studies for inclusion and the *a priori* framework are identified. The identification and selection of primary studies for inclusion in the review and the identification and generation of the *a priori* framework occur simultaneously but independently. These two strands then join together at the second stage where the synthesis process begins.

The two-stage seven-step method of “best fit” framework synthesis that will be followed in this review is described in detail below (See Figure 1). The terminology adopted throughout the protocol is consistent with existing literature ^29^ and published examples of “best fit” framework synthesis ^31^. The systematic review and synthesis of the findings will be completed and reported in accordance with the Preferred Reporting Items for Systematic Reviews and Meta-Analyses (PRISMA) ^32^ and the Enhancing Transparency in Reporting the Synthesis of Qualitative Research (ENTREQ) ^33^ reporting guidelines.

### Review question (Stage 1 – Step 1)

A scoping search was conducted to explore the amount and nature of the evidence and to inform the review aim and question. The following review question was developed using the SPIDER (Sample, Phenomenon of Interest, Design, Evaluation, Research Type) framework ^34^:

• What are the barriers and enablers perceived by adults with type 2 diabetes to sustaining self-management behaviours after completing a diabetes self-management support intervention?

### Eligibility criteria

The following eligibility criteria were informed by the SPIDER framework ^34^.

*Sample*. Studies including adults (aged ≥ 18 years) with a diagnosis of type 2 diabetes who have attended a self-management support intervention will be included. If the study also contains participants with type 1 diabetes, individuals with type 2 diabetes aged under 18 years, women with gestational diabetes or adults with type 2 diabetes who did not attend a self-management support intervention, studies will be included only if it is possible to extract the barriers and enablers perceived by the relevant participants.

*Phenomenon of interest*. Studies will be included if they focus on barriers and enablers to sustaining self-management behaviours at least three months after completing a self-management support intervention. For the purpose of this review, a barrier is defined as any factor that impedes or obstructs sustaining self-management behaviours. An enabler is defined as any factor that facilitates or helps sustain self-management behaviours. Consistent with a previous review ^14^, a self-management support intervention for type 2 diabetes is defined as any intervention that aims to support or facilitate self-management of type 2 diabetes. In accordance with the available literature on self-management support for type 2 diabetes, interventions should explicitly focus on self-management support of type 2 diabetes and target one or more of the following self-management domains to be included in the review ^17, 35^:

Cognitive skills

- Education about the disease process, progression, management, and treatments available.
- Goal setting to promote health and facilitate health behaviour change.
- Empowerment or self-efficacy.

Behavioural skills

- Nutritional education and management.
- Physical activity.
- Medication intake.
- Blood glucose monitoring.
- Prevention, management, and treatment of health complications.

Emotional skills

- Psychosocial adjustment.
- Distress, anxiety, and depression management.
- Social support.

There will be no restrictions for inclusion based on the intervention setting, mode of delivery, type of facilitator, intensity, duration, and theoretical basis of the intervention. Studies that include carers or family relatives will be included in the review, as long as the intervention is primarily intended for the person with type 2 diabetes. If the study includes both individuals still attending the intervention and individuals who have completed the intervention, studies will only be include if it is possible to extract data from individuals who completed the intervention and have experiences of self-management for at least three months post-intervention at the time of the study. If studies explore sustaining self-management behaviours and other concept(s) in self-management, it must be possible to extract the information specific to sustaining self-management behaviours to be included in the review. Studies examining other aspects of living with type 2 diabetes (e.g., coping, emotional distress) where it is impossible to extract the data on barriers and enablers to sustaining self-management behaviours after completing a self-management support intervention will be excluded.

*Design and Research Type*. Primary qualitative and mixed-methods research studies will be considered for inclusion. Although grey literature can be difficult to search and retrieve, non-peer reviewed studies will be considered for inclusion as there is a growing consensus that the inclusion of grey literature can widen the scope of reviews, thus providing a more complete picture of the evidence available ^36, 37^. Studies will be included if a qualitative method is used for data collection (e.g., focus groups or interviews) and analysis (e.g., thematic analysis or grounded theory). The data collection and analysis methods should be clearly reported in the studies to be included in the review. Studies that collect data using qualitative methods but analyse it quantitatively (e.g., descriptive statistics) will not be included in the review. Mixed-methods studies will be included in the review if the qualitative data is relevant and of sufficient depth to be synthesised in the review. Only articles published in English will be included in the review. Quantitative studies, literature reviews, qualitative evidence synthesises, editorials, commentaries, opinion pieces, and abstracts in proceedings will be excluded. Where the full text article is not available online, the corresponding author(s) will be contacted by email with one follow-up. If the corresponding author(s) do not reply within one week after the follow-up, the article will be excluded.

### Information sources and search strategy (Stage 1 – Step 2a)

A combination of systematic searching of the literature of electronic databases and supplementary searching techniques will be used to maximise the identification of relevant papers for inclusion in the review ^34^. A comprehensive search will be conducted on the following databases: MEDLINE (Ovid), EMBASE (Elsevier), CINAHL (EBSCO), PsycINFO (Ovid) and SCOPUS from inception to September 2021. An expert librarian provided support on the selection of the databases and development of the search strategy.

The search strategy was informed by the SPIDER framework ^34^ (See Table 1 for further details) in consultation with an information specialist. The search combines free-text terms with index terms (e.g., Medical Subject Headings) for type 2 diabetes, self-management support interventions, sustained behaviour change, and qualitative research. The search strategy was developed iteratively and informed by existing reviews ^14, 19, 38– 41^ to ensure the search was as comprehensive as possible. Methodological filters for qualitative research were also used where available in specific databases to enhance the specificity of the search ^42, 43^. Search terms were truncated where relevant to ensure all spellings are captured (e.g., behavio*). In addition, Boolean terms, such as OR and AND were included in the search to enhance specificity and sensitivity. A sample search strategy for the MEDLINE (Ovid) database is presented in *Extended data:* Supplementary File 3 ^28^.

**Table 1.** Inclusion criteria.

SPIDER	Description
S: Sample	Adults aged 18+ with a diagnosis of type 2 diabetes mellitus
P of I: Phenomenon of interest	Barriers and enables to sustained self-management behaviour change after completing a diabetes self-management support intervention
D: Design	Qualitative research methods (data collection and analysis), including interviews, focus groups, case study, observational study, grounded theory, phenomenology, ethnography, thematic analysis, constant comparison, open-ended questions, content analysis, themes, category
E: Evaluation	Experiences, views, perspectives, perceptions, beliefs, opinions, barriers and enablers or facilitators
R: Research	Primary qualitative studies of any design and mixed-methods that report qualitative findings separately

A search of grey literature will also be undertaken on ProQuest Dissertations and Theses, WorldCat via the Online Computer Library Center (OCLC) and Open Grey in September 2021. To counteract common challenges in identifying qualitative literature through systematic searching of electronic databases alone ^37^, forward, and backward citation searches will be conducted on all included studies.

### Screening (Stage 1 – Step 3a)

The lead author will import all references to the electronic reference manager EndNote X20 and remove duplicates. The eligibility criteria will be pilot tested with a random sample of 6 papers by two authors (MC and PD) and the criteria clarified if needed. The lead author will then screen the titles and abstracts and full texts of the identified articles against the eligibility criteria using Rayyan QCRI software ^44^. A second author (PD) will independently screen a random sample of 20% of the articles at both stages. A chance-corrected Kappa statistic will be calculated to assess inter-reviewer agreement ^45^ at both stages. Disagreements will be discussed between the authors and, if necessary, with a third author until consensus is achieved. When the abstract is not available or does not contain enough information to make an informed decision about the inclusion of an article, the article will be retrieved for full text screening. If necessary, authors of primary studies will be contacted for clarification and further information. All the studies identified as potentially relevant by one or both authors will be retrieved for full text screening. A table listing studies excluded from the review and the main reasons for exclusion will be recorded by the research team and presented in the review using a Preferred Reporting Items for Systematic Reviews and Meta-Analyses (PRISMA) flow diagram ^32^.

### Data extraction and management (Stage 1 – Step 3a)

Full-text articles will be imported to QSR International’s NVivo v12 software ^46^. This software will be used to store the data and to assist in the data extraction and synthesis to ensure clarity and transparency ^47^. A data extraction form (See *Extended data*: Supplementary File 4) will be created for the purpose of this synthesis within NVivo ^47, 48^. The following data will be extracted from each study ^48^: study information (e.g., authors, year of publication), study characteristics (e.g., aims and objectives, sample size), participants’ characteristics (e.g., age, gender, time living with the diagnosis), intervention characteristics (e.g., intervention components, duration, and mode of delivery), data collection and analysis methods. Findings, including participants’ verbatim quotes and reported interpretations by the primary study authors will be extracted as “best fit” framework synthesis allows the analysis, synthesis and integration of primary and secondary data ^31, 49^. Reported strengths, limitations and implications of the study will also be extracted.

The data extraction form will be initially pre-tested with a random sample of three papers by two authors (MC and PD). One author (MC) will extract data from all included articles and a second author (PD) will cross-check 20% of the articles to ensure consistency and minimise potential bias during the data extraction process. Any disagreement will be discussed between the authors and, if necessary, with a third author until consensus is achieved.

### Assessment of methodological limitations of the included studies (Stage 1 – Step 3a)

An adapted version of the Critical Appraisal Skills Programme (CASP) Tool ^50^ for qualitative studies will be used to assess the methodological limitations of the included studies. The following domains will be considered: context, sampling strategy, data collection, data analysis, support of individual study findings in data, reflexivity, and ethical considerations. Each domain will be judged as yes (i.e., the domain is sufficiently, clearly, and appropriately described in the study), no (i.e., the domain is not described in the study) or unclear (i.e., the study only offers a limited or unclear description of the domain). MC will appraise all the included studies. PD will independently appraise 20% of the included studies. Disagreements will be discussed, and a third author will be consulted if necessary. Studies will not be excluded based on this assessment ^51^, in line with recommendations ^52^, but this information will be considered in the analysis of the findings, assessment of confidence in the review findings and the reporting of the review.

### Identification and development of the a priori framework (Stage 1 – Steps 2b and 3b)

The *a priori* framework for this review will be selected through a combination of literature search, expert consultation, and research team consensus ^29^. Potential *a priori* theories will be identified by the review team opportunistically from within the topic-relevant searches and articles selected for full text screening, and purposively from an independent parallel search in Google Scholar combining the search terms ‘model*’ or ‘framework*’or ‘theoretical’ or ‘theory’ or ‘concept’ or ‘conceptual’ ^31^ with the terms ‘type 2 diabetes self-management’ and ‘sustained behaviour change’. A list of relevant candidate theories will be developed based on the results of this search. This approach has been previously used to identify an appropriate existing conceptual model in a worked example of “best Fit” framework synthesis ^31^. Google Scholar has the advantages of covering mainstream and non-mainstream academic literature and ability to sort the results of the search by relevance. Records retrieved will be read to identify relevant candidate theories. The aim is to build a comprehensive list of candidate theories. For each new record, the theory or theories identified will be matched to ones previously identified and added to the list if not previously identified. The search will be terminated when no new theories are identified after five new records have been read ^53^.

As it is anticipated that the number of hits for the initial search will be high with a large number of records of very low relevance, the search results will be ordered by relevance using the Google algorithm ‘sort by relevance’ ^53^.

When a list of potential theories is identified, the review team will meet to discuss the suitability of the candidate theories, and to determine if a single comprehensive theory can be used, or if it is necessary to develop a meta-framework using concepts or constructs from different theories in the existing literature ^49^. The list of candidate theories will be circulated by the research team in advance of the meeting. Additionally, the three senior research team members (DK, MB, JMS) with extensive knowledge of and experience using behavioural theories will be consulted to identify any potentially relevant additional theories they are aware of which are not included in it the list.

The review team will discuss the conceptual fit of each of the candidate theories until consensus is achieved. The three criteria outlined by Damschroder and colleagues ^54^ will be considered by the review team when evaluating candidate theories, as suggested by Booth and Carroll ^49^ (See Table 2). The assumption by Booth and Carroll ^49^ that *a priori* theoretical framework does not need to be “a perfect match for the question or evidence”, but only offer “a ‘good enough’ starting point as designated by the phrase “best fit” (p. 701) will be taken into consideration by the research team. After identifying the most suitable theory, secondary thematic analysis ^29^ will be employed to create the *a priori* framework. Thematic analysis will be used to generate a set of explanatory constructs and theoretical propositions, referred to in the “best fit” synthesis method as themes, which represent patterns of theoretical explanations. The theoretical themes identified might be further organised by subthemes if appropriate.

**Table 2.** Criteria outlined by Damschroder and colleagues ^54^ to evaluate candidate theories.

Criteria	Description
Clarity and coherence of the terminology used by the framework	External validity: Are the concepts readily understandable to the research team? Internal validity: Can the concepts be consistently operationalised by different authors?
Transferability	Does the framework enable comparison of results across studies?
Room for new theoretical developments	Does the framework allow new theoretical developments?

Based on the information available in the primary studies and original papers of the selected theory, definitions will be created for each theme ^29^. As the suitability of a theory or theories also depends on the proportion of the data that can be accommodated within it, the choice of the *a priori* framework will be revisited during the data analysis and synthesis process to ensure the framework selected is the most appropriate ^29^. The *a priori* framework will be considered appropriate if it accommodates at least 50% of the data extracted from the included primary studies.

### Data analysis and synthesis (Stage 2 – Steps 4 to 8)

The lead author will develop a coding tree on NVivo with the themes and constructs identified to facilitate the coding of the data against the *a priori* framework. A second author (PD) will cross-check the final list of themes to ensure different authors can consistently code data from primary studies with a sample of three studies ^29^. The findings from the included studies will be coded against the themes generated based on the *a priori* framework (Step 4). New themes will be generated to code data that cannot be coded against the *a priori* framework secondary thematic analysis ^29^. New themes will be based on the author’s interpretation of the data and constant comparison of such data across studies ^29^ (Step 5). The new themes resulting from this analysis will be added to the *a priori* framework.

A new updated framework composed of *a priori* and new themes supported by the evidence will result from this process ^29, 49^ (Step 6). One author (MC) will conduct all stages of data analysis and synthesis from coding to interpretation with continuous input from the rest of the research team. A second author (PD) will independently analyse and synthesise 20% of the articles. Any discrepancies will be discussed between the two authors and if necessary, with a third author until consensus is achieved.

The potential ways in which themes may relate to each other will then be explored using constant comparison method ^55, 56^, facilitating the generation of a new conceptual model describing the process of sustaining type 2 diabetes self-management behaviours after completing a self-management support intervention ^29, 49^ (Step 7).

Finally, a sensitivity analysis ^29, 57^ will be performed to examine the contribution of studies with methodological limitations to the review findings ^29, 47, 57^ (Step 7). Subgroup analyses will be conducted where appropriate and if possible, comparing studies based on intervention characteristics, time gap between the completion of the intervention and data collection, participants’ characteristics, and study context.

### Confidence in the review findings

The Confidence in the Evidence from Reviews of Qualitative Research (GRADE-CERQual) ^58^ approach will be used to assess confidence in each theme included in the final model. GRADE-CERQual assesses confidence in review findings, based on the following key components: methodological limitations (i.e., the extent to which there are concerns about the design or conduct of the primary studies that contributed to the review finding); relevance (i.e., the extent to which findings from the primary studies are relevant to the review question), coherence (i.e., the extent to which the review findings are grounded in data from the primary studies), and adequacy of the data (i.e., the extent to which a review findings are supported by rich data and a large number of studies) ^58^. After assessing each of the four components, a judgement about the overall confidence in the review finding will be made. The confidence in each review finding will be graded as high (i.e., it is highly likely), moderate (i.e., it is likely), low (i.e., it is possible) or very low (i.e., it is unclear) dependant on whether the review finding is judged to a reasonable representation of the phenomenon of interest ^58^. All findings will initially be graded as high confidence and will then be graded down if there are important concerns regarding any of the GRADE-CERQual components ^58^. The final assessment will be based on consensus among the two authors (MC and PD) involved in the confidence assessment with discussion with the full review team if needed.

### Reflexivity

To ensure rigour and quality, the research team members will maintain a reflexive stance throughout all stages of the review process, from study selection to data synthesis and interpretation of the findings. A team-based reflexive approach will be adopted involving individual critical reflection on assumptions and potential biases and reflexive group discussions ^59^. The research team will continuously reflect on their background and how their personal views and beliefs could influence their choices regarding methods to use, data extraction, coding synthesis, and interpretation of the review findings. MC, JMS, DK and MB have a background in Health Psychology and primarily work in research focused on health behaviour change. MB is a professor, JMS a lecturer, DK is a senior research fellow, and MC is a PhD candidate. PD is a PhD candidate with a background in nutrition/dietetics and has extensive clinical experience delivering self-management support interventions for people with Type 2 diabetes. MB and JMS have experience in conducting qualitative evidence synthesis and primary qualitative research focused on type 2 diabetes mellitus. MC and PD have previous experience in conducting primary qualitative research. DK has experience and expertise in theory review and synthesis. All authors believe that sustaining self-management behaviours is challenging, and people might struggle to integrate these behaviours into their daily lives.

During the screening, data extraction, coding and synthesis, and assessment of confidence in the review findings, the team will regularly meet to discuss progress and potential disagreements. The two authors (MC and PD) who will conduct the study screening, data coding, extraction and synthesis, and assessment of confidence in the review findings, will meet regularly to discuss how their background, experiences and presumptions on the review topic may be influencing their assessments and analysis and will both record and reflect on their decisions in memos. As the lead author, MC will keep a reflexive journal throughout the review process to document and critically reflect on the research process.

### Public and Patient Involvement

The involvement of key stakeholders in systematic reviews is increasingly recognised as fundamental to the quality, relevance, and impact of the review findings ^60, 61^. Patient and public representatives of people with type 2 diabetes who attended a self-management support intervention for type 2 diabetes will be involved in the review from the point of data synthesis. Patient and public representatives will be asked to review the *a priori* framework and the new themes that are generated by the research team and contribute to the new updated framework and interpretation of the synthesis findings. In addition, they will also be invited to contribute to the development of dissemination strategies and assist in the preparation of dissemination documents, such as the lay summary, to ensure clarity and readability. The involvement of the members from the advisory panel in the systematic review process will be guided by the ACTIVE (Authors and Consumers Together Impacting on eVidencE) framework, which outlines a range of methods and approaches to guide both the involvement of stakeholders in systematic reviews and the reporting of their involvement in the review process ^61^. The activities and contributions of the advisory panel will be reported in line with the Guidance for Reporting Involvement of Patients and the Public Version 2 (GRIPP2) Checklist ^62^ and the ACTIVE framework ^61^.

### Study status

The review is currently underway. The database searches have been completed, duplicates removed, title and abstract screening completed, and full text screening commenced.

## Discussion

Given the decline in glycaemic control over time following completion of a self-management support intervention, there is a need to better understand ‘how’ (and why) people self-manage their diabetes post-interventions. This review will be the first to explore barriers and enablers experienced by people with type 2 diabetes to sustaining self-management behaviours after completing a self-management support intervention. By adopting the “best-fit” framework synthesis method, the review will result in a comprehensive model of the maintenance of type 2 diabetes self-management behaviours after completion of a self-management support intervention. The model is anticipated to identify factors that influence the self-management of type 2 diabetes over time and might contribute to the variability in the long-term effectiveness of this type of interventions ^14^. The evidence-informed conceptual model resulting from this review will be useful to guide future intervention revision or design. In addition, the model resulting from this review will provide important theoretical insights into the process of sustained behaviour change, a key priority area in behavioural science ^19^.

The planned review has several strengths and limitations. The review methods are transparent, rigorous and will be reported in accordance with published guidelines ^27, 32, 33^. An audit trail detailing the decisions made and methodological steps taken will be kept throughout the research process. Due to practical reasons, we will not include non-English articles whose findings could provide interesting additional insights. Despite the best efforts of the research team, it is also possible that not all relevant articles will be retrieved during the search for primary studies and/or selected during the screening process due to the myriad of terms used in the literature to describe self-management support interventions, sustained self-management and qualitative research. This review will also reflect the limitations of the included studies as the review findings will be limited to what is reported in the included primary studies. Another potential limitation relates to the heterogeneity of the self-management support interventions described in the primary studies and the time gap between the completion of the intervention and data collection. Differences between interventions and the amount of time since the completion of the programme have potential to make the synthesis across studies and the drawing of appropriate conclusions more difficult.

## Dissemination

Findings from the review will be submitted to a peer-reviewed journal for publication and the final review results will be promoted in social media outlets, including Twitter, to reach a wider public audience. The findings will also be disseminated to key stakeholders at relevant national and international conferences, and a policy brief and a lay summary will be created to communicate the findings to policymakers, people with type 2 diabetes and the general public. In addition, alternative dissemination strategies suggested by the members of the public and patient advisory panel will also be considered.

## Data availability

### Underlying data

No data are associated with this article. However, dataset associated with the review will be published Open Access online on the Open Science Framework review page.

### Extended data

Open Science Framework: SUSTAIN T2DM: Supporting people with type 2 diabetes to sustain self-management behaviours, https://doi.org/10.17605/OSF.IO/KJVGU ^28^.

The project contains the following extended data (under Work Package 1: Barriers and enablers to sustaining self-management behaviours after completing a self-management support intervention for type 2 diabetes: A systematic review and qualitative evidence synthesis):

- Supplementary file 2 - RETREAT Framework.docx
- Supplementary file 3 – MEDLINE Search Sample.doc
- Supplementary file 4 - Data Extraction Form.doc

### Reporting guidelines

Open Science Framework: Supplementary File 1. PRISMA-P checklist, https://doi.org/10.17605/OSF.IO/KJVGU ^28^.

Data are available under the terms of the Creative Commons Zero "No rights reserved" data waiver (CC0 1.0 Public domain dedication).
