# Supplementary material for: Barriers and enablers to sustaining self-management behaviours after attending a self-management support intervention for type 2 diabetes: a protocol for a systematic review and qualitative evidence synthesis
Source: HRB Open Res. 2022 Jun 13;4:129. Originally published 2021 Dec 14. [Version 2] doi: 10.12688/hrbopenres.13466.2 (PMC8886171; doi:10.12688/hrbopenres.13466.2)
Supplement: Peer review history

# Peer review: Reviewer response for version 2

**DOI:** 10.21956/hrbopenres.14822.r32307

**Published:** 2022-06-13

**Authors:** Pauline Meskell

**Affiliations:** 1 Department of Nursing and Midwifery, University of Limerick, Limerick, Ireland

Highlighted areas for review have been addressed appropriately.

Is the study design appropriate for the research question?

Yes

Is the rationale for, and objectives of, the study clearly described?

Yes

Are sufficient details of the methods provided to allow replication by others?

Yes

Are the datasets clearly presented in a useable and accessible format?

Yes

Reviewer Expertise:

Qualitative Evidence Synthesis methodology

I confirm that I have read this submission and believe that I have an appropriate level of expertise to confirm that it is of an acceptable scientific standard.

# Peer review: Reviewer response for version 1

**DOI:** 10.21956/hrbopenres.14677.r31087

**Published:** 2022-02-28

**Authors:** Pauline Meskell

**Affiliations:** 1 Department of Nursing and Midwifery, University of Limerick, Limerick, Ireland

The protocol gives background and context to the issues around the complexities of sustaining self management behaviors for individuals living with T2 Diabetes Mellitus. People that undergo educational interventions in self management often do not sustain the behaviors long term and this can have negative consequences on their overall health and diabetes management, The aim of the review was to synthesis qualitative evidence on the barriers and facilitators  to sustaining self management behaviors following completion of a self management support intervention for T2DM.

 The review authors detail the planned methodology of the QES using a Best Fit Framework synthesis approach. Good detail is provided in relation to the development of the review question, and the intended format of the best fit framework approach.  Clear descriptions of the criteria is outlined. The design and research type of studies is well detailed. No justification of why only articles in English is given, this is a potential limitation of the review. Appropriate level details is included for searching and screening both for the review and the best fit framework.  The approach to identify a best fit framework appears particularly complex, and would appear to complicate the process of apriori coding rather than facilitate it. If the framework is sufficiently well developed it should not require secondary thematic analysis and perhaps the choice of framework needs to be cognizant of this.

 The subgroup analysis could include socioeconomic factors, such as income level of country where research was carried out as this may have an influence on outcomes of a self management intervention. Good reflexive account of review authors positions in the topic area and review.  Inclusion of PPI is a positive step towards meaningful inclusion of patients/public.

 Overall a well detailed protocol, that evidences good understanding of the processes and requirements of a QES.

Is the study design appropriate for the research question?

Yes

Is the rationale for, and objectives of, the study clearly described?

Yes

Are sufficient details of the methods provided to allow replication by others?

Yes

Are the datasets clearly presented in a useable and accessible format?

Yes

Reviewer Expertise:

Qualitative Evidence Synthesis methodology

I confirm that I have read this submission and believe that I have an appropriate level of expertise to confirm that it is of an acceptable scientific standard.

# Response

**Published:** 2022-06-01

**Authors:** Márcia Carvalho

**Affiliations:** National University of Ireland, Galway, Ireland

**C3.1. The protocol gives background and context to the issues around the complexities of sustaining self management behaviors for individuals living with T2 Diabetes Mellitus. People that undergo educational interventions in self management often do not sustain the behaviors long term and this can have negative consequences on their overall health and diabetes management, The aim of the review was to synthesis qualitative evidence on the barriers and facilitators  to sustaining self management behaviors following completion of a self management support intervention for T2DM.**

*R3.1.:* We thank the reviewer for the comments on our manuscript.

**C3.2. The review authors detail the planned methodology of the QES using a Best Fit Framework synthesis approach. Good detail is provided in relation to the development of the review question, and the intended format of the best fit framework approach.  Clear descriptions of the criteria is outlined. The design and research type of studies is well detailed. No justification of why only articles in English is given, this is a potential limitation of the review. Appropriate level details is included for searching and screening both for the review and the best fit framework.**

*R3.2.:* The research team agrees that the inclusion of articles in English only is a limitation of the review and has reflected on the potential impact of this decision in the discussion section of the protocol.

 The team decided to only include studies in English to avoid issues with language translation of qualitative studies as meaning may be lost in translation (van Nes et al., 2010). This decision was also made for practical reasons, including limited access to translation services and a limited timeframe as this review is being conducted as part of a wider PhD project. The protocol has been amended to clarify why only articles in English will be included. The following sentences and reference have been added to page 7 (phenomenon of interest):

 “Only articles published in English will be included in the review to avoid issues with language translation of qualitative studies as meaning may be lost in translation ^38^, as well as pragmatic constraints including a limited timeframe for the review, and limited access to translation services”.

 We will also reflect on this limitation in the final manuscript reporting the findings of the review.

**C3.3. The approach to identify a best fit framework appears particularly complex, and would appear to complicate the process of a priori coding rather than facilitate it. If the framework is sufficiently well developed it should not require secondary thematic analysis and perhaps the choice of framework needs to be cognizant of this.**

*R3.3.:* We have decided to be cautious in the protocol and cover all possibilities in relation to developing the a priori framework including the need for secondary thematic analysis. However, it is anticipated that the a priori framework chosen to guide data coding will be developed enough that it will not require secondary thematic analysis.

**C3.4. The subgroup analysis could include socioeconomic factors, such as income level of country where research was carried out as this may have an influence on outcomes of a self-management intervention.**

*R3.4.:* The protocol has been amended to add further details on the subgroup analysis in response to the suggestion of the reviewer. We have rephrased the last sentence of the data analysis and synthesis section (page 12) to read:

 “Subgroup analyses will be conducted where appropriate and if possible, comparing studies based on intervention characteristics, the time gap between the completion of attendance at the intervention and data collection, participants’ characteristics, and study context, including continent and income level of the country where the study was carried out”.

**C3.5. Good reflexive account of review authors positions in the topic area and review.  Inclusion of PPI is a positive step towards meaningful inclusion of patients/public.**

**Overall a well detailed protocol, that evidences good understanding of the processes and requirements of a QES.**

*R3.5.: *We thank the reviewer for the comments on our manuscript.

# Peer review: Reviewer response for version 1

**DOI:** 10.21956/hrbopenres.14677.r31171

**Published:** 2022-02-01

**Authors:** Angela M. Rodrigues

**Affiliations:** 1 Department of Psychology, Northumbria University, Newcastle upon Tyne, UK

This paper is an important addition to the literature that will summarise qualitative research on the barriers and enablers to sustaining self-management behaviours. I really like the potential of generating a comprehensive theoretical framework of sustained behaviour change in type 2 diabetes self-management as a result of this work. The paper also provides an excellent description of this systematic review and qualitative evidence synthesis methodology. Some very minor comments below:

- Sensitivity analysis – Further elaboration might be beneficial. Can you detail how exactly you are going to conduct the sensitivity analysis? From your writing, I can sense that you know exactly how you are going to do it but might be worth spelling it out. For readers less familiar with sensitivity analyses in narrative synthesis this information might be particularly relevant.
- Reflexivity – I really like this section and find it very innovative to add this to a narrative synthesis. Some further details might be needed here. How exactly are you going to do this? Are you going to keep a reflective logbook for every part of the process, for instance? How will you integrate this into your interpretation of the findings?
- Publication plan – If possible/known, it might be appropriate to specify how many papers you anticipate publishing as a result of this work.

Is the study design appropriate for the research question?

Yes

Is the rationale for, and objectives of, the study clearly described?

Yes

Are sufficient details of the methods provided to allow replication by others?

Partly

Are the datasets clearly presented in a useable and accessible format?

Yes

Reviewer Expertise:

Diabetes prevention, behavioural science, systematic reviews

I confirm that I have read this submission and believe that I have an appropriate level of expertise to confirm that it is of an acceptable scientific standard.

# Response

**Published:** 2022-06-16

**Authors:** Márcia Carvalho

**Affiliations:** National University of Ireland, Galway, Ireland

**C2.1. This paper is an important addition to the literature that will summarise qualitative research on the barriers and enablers to sustaining self-management behaviours. I really like the potential of generating a comprehensive theoretical framework of sustained behaviour change in type 2 diabetes self-management as a result of this work. The paper also provides an excellent description of this systematic review and qualitative evidence synthesis methodology. Some very minor comments below:**

*R2.1.:* ****We thank the reviewer for the comments on our manuscript.

**C2.2. Sensitivity analysis – Further elaboration might be beneficial. Can you detail how exactly you are going to conduct the sensitivity analysis? From your writing, I can sense that you know exactly how you are going to do it but might be worth spelling it out. For readers less familiar with sensitivity analyses in narrative synthesis this information might be particularly relevant.**

*R2.2.:* ****As suggested, we have added the following sentences to the protocol (see page 9 data analysis and synthesis) to provide further details on how we will conduct the sensitivity analysis:

 “The query tools within NVivo, such as matrix-coding queries, will be used to conduct the sensitivity analysis following the guidance provided by Houghton and colleagues (2017) ^48^. Matrix-coding queries allow for the comparison of multiple nodes/codes and attributes as a numeric matrix table with different shading colours, which indicates whether studies with methodological limitations skewed the findings in any particular way based on their contribution to the finding ^48^. Each study will be assessed for methodological limitations based on the CASP tool criteria to facilitate this analysis ^51^”.

**C2.3. Reflexivity – I really like this section and find it very innovative to add this to a narrative synthesis. Some further details might be needed here. How exactly are you going to do this? Are you going to keep a reflective logbook for every part of the process, for instance? How will you integrate this into your interpretation of the findings?**

*R2.3.*: To ensure we keep a reflective stance throughout the review, the two review authors involved in screening, data extraction and synthesis (MC and PD) have engaged in writing individual reflexive diaries and field notes, which have been recorded in memos on NVivo and form the basis for ongoing discussion in research team meetings. In addition, the two authors have been using the seven questions proposed by Barry et al. (1999) to guide their reflexive accounts. Examples of questions proposed by Barry et al. (1999) include: ‘In what way might my experience colour my participation in the project?’, ‘What is my orientation to qualitative research?’, and ‘What results do I expect to come out of this project?’. The two authors have reflected on these questions and recorded their thoughts at two-time points so far: at the start of the review before commencing screening, and in the middle of the review before commencing data synthesis. The authors’ reflections were recorded in memos on NVivo.

 By keeping this reflexive stance, we hope to reduce potential threats to rigour and to articulate any potential impact of personal or professional beliefs or biases on the interpretation of the findings. The team has been considering and discussing any potential conflicts of interests, or biases, carefully as a team and keeping records of reflexive accounts and decisions made throughout the research process, including the rationale for the decisions. As suggested by Flemming et al. (2017), the following aspects related to author reflexivity will be reported on the final manuscript of the review: (a) the funder and their potential involvement and influence on the development and editing of the review findings; (b) the composition of the review team and the key positions or beliefs about the review question and the phenomenon of interest that could influence the interpretation of the findings; (c) potential conflicts of interest both financial and non-financial; (d) team governance procedures and processes to ensure internal validity; (e) procedures for processing evidence when one of the review authors is also an author of a primary qualitative study; and (f) procedures and processes to engage with key stakeholders ensuring no potential undue influence on the review and interpretation of the findings. The protocol has been amended as per below to provide further details on the author’s reflexivity and clarify the queries raised by the reviewer on page 9 of the protocol (reflexivity):   

 “Both MC and PD will keep reflexive diaries and field notes, which will be recorded in memos. The authors will use the questions proposed by Barry et al. (1999) ^60^ to guide their reflexive accounts. Examples of questions proposed by Barry et al. (1999) ^60^ include: (1) ‘in what way might my experience colour my participation in the project?’, (2) ‘what is my orientation to qualitative research?’, and (3) ‘what results do I expect to come out of this project?’. By keeping this reflexive stance, the research team hopes to identify potential threats to rigour and any potential negative impact of any personal or professional beliefs or biases on the interpretation of the findings.

 As suggested by Flemming and Noyes (2017) ^61^,  the following aspects relevant to author reflexivity will be reported on the final manuscript of the review: (a) the funder and their potential involvement and influence on the development and editing of the review findings; (b) the composition of the review team and the key positions or beliefs about the review question and the phenomenon of interest that could influence the interpretation of the findings; (c) potential conflicts of interest both financial and non-financial; (d) team governance procedures and processes to ensure internal validity; (e) procedures for processing evidence when one of the review authors is also an author of a primary qualitative study; and (f) procedures and processes to engage with key stakeholders ensuring no potential undue influence on the review and interpretation of the findings ^61”^.

**C2.4. Publication plan – If possible/known, it might be appropriate to specify how many papers you anticipate publishing as a result of this work.**

*R2.4.:* ****We anticipate publishing only one paper based on this review. This has been specified in the dissemination section on page 10 of the manuscript to enhance clarity.

# Peer review: Reviewer response for version 1

**DOI:** 10.21956/hrbopenres.14677.r31085

**Published:** 2022-01-31

**Authors:** Leah Avery

**Affiliations:** 1 Translational and Clinical Research Institute, Newcastle University, Newcastle upon Tyne, UK; 2 Centre for Rehabilitation, School of Health & Life Sciences, Teesside University, Middlesbrough, UK

The aim of this systematic review is to synthesise qualitative research on the barriers and enablers to sustaining self-management behaviours following the completion of a self-management intervention for type 2 diabetes.

 It is widely accepted that people with type 2 diabetes experience difficulties in making and sustaining lifestyle behaviour changes to effectively manage their condition. The authors highlight this point, with specific reference to sustaining changes, which provides a strong rationale for undertaking the systematic review. A number of systematic reviews exist that report on the effectiveness of self-management interventions, however, there is a lack of specific evidence reporting on the barriers and enablers. This context is vitally important to optimise existing interventions, to inform the development of new interventions, and in the case of this piece of research, to develop models of sustained behaviour change.  The study is clearly described in terms of subject matter, methods, and intended outcomes, and in my view will make a valuable and much needed contribution to the field. I do, however, have a few queries as follows:

 The study design is appropriate for the research question, although I have a number of queries regarding the eligibility criteria. The review aims to include qualitative studies and multi-methods studies that report qualitative findings separately. Does that refer to a separate (but linked) publication or a separate section of the mixed-methods publication, or both? As such, would this include a publication reporting on a randomised controlled trial of an intervention with a qualitative sub-study, as long as the findings from the sub-study were reported in sufficient detail within the same paper? I see that the authors have made reference to ‘sufficient detail’, but in the case of the two situations I have described, the level of detail could be very different. Could the authors perhaps provide further information on what will be sufficient?

 In terms of sustained behavioural change at 3-months post-intervention, how will this be verified in a situation where a publication reporting on a primary qualitative study is retained for review?

 By sustained, are the authors referring to any change from baseline sustained or change from completion of the intervention sustained (e.g., if a person increases physical activity by 50 minutes per week at the 1-month intervention time point and an additional 30 minutes per week at the 2-month intervention time point, will the changes be considered as ‘sustained’ if 20 minutes per week is reported upon intervention completion at 4-months)? I appreciate this relates to definitions of maintenance, however, it would be useful to clarify for the purpose of this review.

 Will the views of non-completers be included in the review if they sustain a change in behaviour (e.g., those who complete 3-weeks of a 3-month intervention but still provide their views)? I ask because the protocol refers to those who have ‘completed’ a self-management intervention, however, I suspect including and synthesising the views of non-completers will be advantageous. It is possible that despite not completing the intervention, any changes made early on could still be sustained.

 The methods reported are very comprehensive and enable replication, specifically in terms of study selection, data extraction, and assessment of methodological quality. Clarification on the queries highlighted in relation to inclusion criteria will further strengthen replicability and augment understanding.

 In relation to the search strategy (page 6), the authors report that methodological filters will be used for qualitative research to enhance specificity where databases provide these filters. While I can understand the merit of doing this, it can sometimes omit relevant studies if the study has not been indexed using the term searched for. It might be worth piloting the search in this regard.

 Overall, this is a robust and well-designed systematic review protocol reviewing literature in a very important area and will likely generate outcomes/resources of significant benefit to the field.

Is the study design appropriate for the research question?

Yes

Is the rationale for, and objectives of, the study clearly described?

Yes

Are sufficient details of the methods provided to allow replication by others?

Partly

Are the datasets clearly presented in a useable and accessible format?

Not applicable

Reviewer Expertise:

Development and evaluation of health behaviour change interventions in the context of type 2 diabetes, NAFLD and other long-term health conditions. Multi-method research/systematic reviews.

I confirm that I have read this submission and believe that I have an appropriate level of expertise to confirm that it is of an acceptable scientific standard, however I have significant reservations, as outlined above.

# Response

**Published:** 2022-06-16

**Authors:** Márcia Carvalho

**Affiliations:** National University of Ireland, Galway, Ireland

**C1.1. The aim of this systematic review is to synthesise qualitative research on the barriers and enablers to sustaining self-management behaviours following the completion of a self-management intervention for type 2 diabetes.**

**It is widely accepted that people with type 2 diabetes experience difficulties in making and sustaining lifestyle behaviour changes to effectively manage their condition. The authors highlight this point, with specific reference to sustaining changes, which provides a strong rationale for undertaking the systematic review. A number of systematic reviews exist that report on the effectiveness of self-management interventions, however, there is a lack of specific evidence reporting on the barriers and enablers. This context is vitally important to optimise existing interventions, to inform the development of new interventions, and in the case of this piece of research, to develop models of sustained behaviour change.  The study is clearly described in terms of subject matter, methods, and intended outcomes, and in my view will make a valuable and much needed contribution to the field. I do, however, have a few queries as follows.**

*R1.1.:* ****We thank the reviewer for the constructive comments on our manuscript.

**C1.2. The study design is appropriate for the research question, although I have a number of queries regarding the eligibility criteria. The review aims to include qualitative studies and multi-methods studies that report qualitative findings separately. Does that refer to a separate (but linked) publication or a separate section of the mixed-methods publication, or both? As such, would this include a publication reporting on a randomised controlled trial of an intervention with a qualitative sub-study, as long as the findings from the sub-study were reported in sufficient detail within the same paper? I see that the authors have made reference to ‘sufficient detail’, but in the case of the two situations I have described, the level of detail could be very different. Could the authors perhaps provide further information on what will be sufficient?**

*R1.2.:* ****Multi-methods or mixed-methods studies that report qualitative findings separately refer to both a separate (but linked) publication or a separate section of the mixed-methods publication. This can include a publication reporting on a randomised controlled trial of an intervention with a qualitative sub-study relevant and of sufficient depth to be synthesised in the review. However, it is important to note that the search retrieved all potentially relevant studies and, therefore, it was not actually necessary to explore separate but linked publications during the screening.

 We considered relevant, and of sufficient depth for inclusion, qualitative data focused on barriers and enablers to sustaining self-management behaviours after attending a self-management support intervention for type 2 diabetes. Studies were included independent of this being the primary or secondary aim of the study, as long as relevant findings were supported by at least one quote from verbatim transcripts. Mixed-methods or multi-method studies that used qualitative data collection methods, but analysed and presented the findings quantitatively (e.g., only code labels and/or descriptive statistics) were not included in the review.

 The protocol has been amended to clarify this. We added the following sentences to the eligibility criteria section (design and research type, page 6):

 “Both mixed and multi-methods studies that report qualitative findings in either a separate linked publication that was cited in the study retrieved in the search or as a separate section of a study retrieved in the search will be considered for inclusion.

 Studies will be included if there is a focus on barriers and enablers to sustaining self-management behaviours after attending a self-management support intervention for type 2 diabetes. Studies will be deemed relevant and of sufficient depth to be synthesised in the review if relevant findings are supported by at least one quote from participants.”

**C1.3. In terms of sustained behavioural change at 3-months post-intervention, how will this be verified in a situation where a publication reporting on a primary qualitative study is retained for review?**

*R1.3.:* ****This has been verified by checking the data collection timing in the study abstract or full text. In many studies, the authors reported the data collection timing (e.g., 6 months post-intervention attendance) in the methods section, specifying the time gap between intervention attendance and data collection. When the authors of the primary studies did not report the time gap between intervention attendance and data collection, the research team contacted the corresponding author(s) for clarification and further information, if contact details were available.

 Studies have been excluded from the review when it is impossible to ascertain the time gap between intervention attendance and data collection in the study. This information has been added to the protocol on page 4 (phenomenon of interest):

 “The data collection timing will be verified in the study abstract or full text to ascertain that data reported in the study were collected at least 3-months post-intervention. If the study is focused on barriers and enablers to sustaining self-management behaviours, but the data collection timing is not reported in the abstract, the study will be retrieved for full-text screening. When the time gap between intervention attendance and data collection is not reported in the study, the research team will contact the corresponding author(s) for clarification and further information. If it is not possible to ascertain the time gap between intervention attendance and data collection, the study will be excluded”.

**C1.4. By sustained, are the authors referring to any change from baseline sustained or change from completion of the intervention sustained (e.g., if a person increases physical activity by 50 minutes per week at the 1-month intervention time point and an additional 30 minutes per week at the 2-month intervention time point, will the changes be considered as ‘sustained’ if 20 minutes per week is reported upon intervention completion at 4-months)? I appreciate this relates to definitions of maintenance, however, it would be useful to clarify for the purpose of this review.**

*R1.4.:* The definition of sustained change is important (and complicated!) both for this study and for the field more generally. For this review, we are focusing on the process of trying to sustain any changes made at least three months after attendance at the intervention, whether that process was successful or not.

 As we are looking at qualitative data, we will focus on participants' perceptions of sustaining long-term change and we will not use a specific definition of whether changes are sustained or not as we do not have access to data on quantitative or objective measures of behaviour change. In the example provided by the reviewer, we would not make a decision as to whether this was sustained change or not but would be interested in the participant’s view of this process, and the barriers and enablers to maintaining this change to physical activity.

**C1.5. Will the views of non-completers be included in the review if they sustain a change in behaviour (e.g., those who complete 3-weeks of a 3-month intervention but still provide their views)? I ask because the protocol refers to those who have ‘completed’ a self-management intervention, however, I suspect including and synthesising the views of non-completers will be advantageous. It is possible that despite not completing the intervention, any changes made early on could still be sustained.**

*R1.5.:* There is a difficulty in some studies in ascertaining if all the participants have completed the intervention (i.e., attended all intervention sessions), particularly in studies exploring the maintenance of randomised control trial outcomes. In these studies, different definitions of intervention completion (e.g., attendance at 75% of the programme sessions) are commonly reported. In other studies, the authors seem to use the terms intervention attendance and completion interchangeably, not specifying how these concepts were defined and/or how many sessions the study participants attended. In addition, as the reviewer highlights, a person who has attended only 50% or 75% of the sessions might also have made changes to self-management behaviours earlier in the intervention which can still be sustained at 3 months post-intervention.

 Therefore, the research team has decided to focus on attendance rather than completion and to consider studies focused on barriers and enablers to sustaining self-management behaviours after attending a self-management support intervention for type 2 diabetes for inclusion in the review. With this change, it is possible that the views of non-completers, or people who did not attend all intervention sessions, will be included in the review. The title of the review and the protocol have been amended to reflect this change.

**C1.6. The methods reported are very comprehensive and enable replication, specifically in terms of study selection, data extraction, and assessment of methodological quality. Clarification on the queries highlighted in relation to inclusion criteria will further strengthen replicability and augment understanding.**

*R1.6.:* The protocol has been amended to provide further clarity on the queries highlighted by the reviewer. These changes are outlined below in response R1.7.

**C1.7. In relation to the search strategy (page 6), the authors report that methodological filters will be used for qualitative research to enhance specificity where databases provide these filters. While I can understand the merit of doing this, it can sometimes omit relevant studies if the study has not been indexed using the term searched for. It might be worth piloting the search in this regard.**

*R1.7.:* ****There is evidence that using validated search filters in qualitative research can result in the omission of potentially relevant studies due to the diversity of terms used to index qualitative research within electronic databases (Booth, 2016). Therefore, we have combined search filters with other synonymous free-text terms in qualitative research to enhance the specificity of the search. We have also piloted the search to ensure the retrieval of all potentially relevant studies (Booth, 2016). The protocol has been amended to reflect to clarify this. The following sentences were added to information sources and search strategy:

 “Using validated search filters in qualitative research can result in the omission of potentially relevant studies due to the diversity of terms used to index qualitative research within electronic databases 37. Therefore, search filters have been combined with other synonymous free-text terms in qualitative research to ensure potentially relevant studies were not missed ^37^”.

**C1.8. Overall, this is a robust and well-designed systematic review protocol reviewing literature in a very important area and will likely generate outcomes/resources of significant benefit to the field.**

*R1.8.:* ****Once again, we thank the reviewer for the constructive comments on our manuscript.
